# From Suitability to Invasion Risk: Integrating Correlative and Mechanistic Models for Predicting the Potential Distribution of the Red Palm Weevil

**DOI:** 10.1002/ece3.74373

**Published:** 2026-09-24

**Authors:** Abdelmutalab G. A. Azrag, Komi Mensah Agboka, Elfatih M. Abdel‐Rahman, Rehemah Gwokyalya, Seid A. Kemal, Hamadttu A. F. El‐Shafie, Mohamed A. Bob, Samira A. Mohamed, Sunday Ekesi, Abdou Tenkouano

**Affiliations:** ^1^ International Centre of Insect Physiology and Ecology (icipe) Nairobi Kenya; ^2^ Laboratoire de Recherche en Science et Technologie (LARSI), Département de Génie Informatique (GI), École Polytechnique de Lomé (EPL) Université de Lomé Lomé Togo; ^3^ International Center for Agricultural Research in the Dry Areas (ICARDA) Rabat Morocco; ^4^ International Center for Agricultural Research in the Dry Areas (ICARDA) Dubai UAE; ^5^ The International Center for Biosaline Agriculture (ICBA) Dubai UAE; ^6^ Department of Zoology and Entomology University of Pretoria Pretoria South Africa

**Keywords:** climate suitability, hybrid modeling framework, invasion risk assessment, palm weevils, *Rhynchophorus ferrugineus*, risk index, species distribution models

## Abstract

The red Palm Weevil (RPW) *Rhynchophorus ferrugineus* is the most devastating pest of palm trees and is responsible for significant economic and biodiversity losses worldwide. The larvae bore deep into the trunk or crown for feeding, resulting in extensive tunneling and mortality of the trees. In this study, we developed a hybrid modeling framework for RPW that integrates ecological niche modeling (ENMs) with a temperature‐driven risk index (RI). The ENM was developed from the RPW occurrence data, while the RI was driven from temperature‐dependent life history traits of RPW. The two approaches were combined through a weighted additive method to enhance predictive performance and biological realism. The correlative SDM achieved the highest predictive performance (AUC = 0.984 ± 0.002; TSS = 0.891 ± 0.009), whereas the mechanistic RI performed substantially lower (AUC = 0.755 ± 0.009 and TSS of 0.457 ± 0.017). Hybrid performance increased with greater SDM contribution, with the 90% SDM–10% RI hybrid (Hybrid90) achieving performance comparable to the SDM (AUC≈0.983 ± 0.002; TSS≈0.893 ± 0.009). Bootstrap analyses showed overlapping 95% confidence intervals between the SDM and Hybrid90 across all evaluation metrics, while the RI remained consistently inferior. Model rankings were also robust across suitability thresholds. Spatial projections revealed high suitability across Southeast Asia, North Africa, the Middle East, the Mediterranean Basin, and coastal Brazil, consistent with known invasion patterns. Additionally, large areas of coastal Africa, Europe, and the Americas were identified as climatically suitable but remain partially uninvaded, indicating substantial invasion risk under the current scenario. Future climate scenarios suggest persistence of current hotspots alongside increased stability and expansion potential, particularly in East and West Africa. Our results demonstrate that integrating correlative and mechanistic information enhances the ecological interpretability of species distribution models without compromising predictive performance. Although the correlative SDM remained the most accurate predictor, the optimal hybrid model achieved comparable performance while incorporating biologically meaningful constraints on species persistence. This transferable framework supports biologically informed invasion‐risk assessment, climate‐change adaptation, and proactive surveillance and management of invasive pests, including the red palm weevil in regions that remain uninvaded.

## Graphical abstract

The invasive *Rhynchophorus ferrugineus* (RPW) causes severe economic and ecological damage to palm systems worldwide. This study develops a hybrid modeling framework integrating correlative ecological niche models and a temperature‐driven mechanistic risk index, demonstrating superior predictive accuracy (sensitivity = 0.98) compared to individual approaches. Results indicate extensive current and future climatic suitability across tropical and subtropical regions, highlighting areas at high risk of invasion and supporting proactive pest management strategies under climate change.

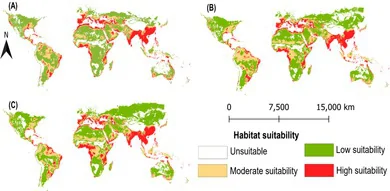

## 1 Introduction

The spread and establishment of invasive insect species in new environments are rapidly increasing worldwide due to global trade and climate change (Seebens et al. 2015; Hulme 2021; Azrag et al. 2023). These species often arrive in new habitats without their co‐evolved natural enemies, leading to high population growth, thus causing significant economic and biodiversity losses (Sileshi et al. 2019; Ndlela et al. 2021). One such pest that has been spreading and causing extensive damage is the Red Palm Weevil (RPW), *Rhynchophorus ferrugineus* (Olivier) (Coleoptera: Curculionidae). RPW is the most devastating pest of palm trees worldwide (Faleiro 2006; Antony et al. 2021; Hoddle et al. 2024). It is native to rainforest of Southeast Asia, but has rapidly expanded its geographical range and established itself through the Middle East, North Africa, Southern Europe, and parts of the Americas, posing a threat to palm cultivation (Nurashikin‐Khairuddin et al. 2022). RPW attacks more than 40 palm species, including date palm, *Phoenix dactylifera* L., coconut, *Cocos nucifera* L., oil palm, *Elaeis guineensis* Jacq., sago palm, *Metroxylon sagu* Rottb., among others (Azmi et al. 2017; Al‐Dosary et al. 2016; Nurashikin‐Khairuddin et al. 2022). A loss of $25.92 million was estimated in the Arabian Gulf region in 2009 due to RPW in date palm, underscoring the economic importance of this pest in the palm industry (El‐Sabea et al. 2009).

Given its destructive potential, RPW requires urgent attention, particularly in palm‐growing regions that have not yet been invaded. Proactive measures, including early detection, risk assessment, and preventive management strategies, are critical for effective management and for reducing the risk of its introduction and establishment in new areas. This can be achieved through modeling the habitat suitability and potential distribution of RPW using species distribution models, which are increasingly recognized as important tools in ecological research and pest risk assessment under changing climatic conditions. These models help identify vulnerable regions that are under high risk of invasion, which in turn aids in designing early warning and management strategies (Karuppannasamy et al. 2024; Azrag et al. 2025).

Two modeling approaches, namely correlative and mechanistic, have been extensively used to predict the potential distribution of many invasive species under current and future conditions (Phillips et al. 2006; Kearney et al. 2010; Shabani et al. 2016). However, these approaches operate on different computational frameworks and analytical principles, with each having its own set of advantages and limitations. Correlative models, also known as ecological niche models (ENM), such as Maxent, random forest, and generalized linear models (GLMs), among others, use statistical or machine learning methods to establish the relationship between the pest occurrence records and environmental variables to compute the conditional probability (Phillips et al. 2006; Shabani et al. 2016; Campos et al. 2023). Although these models are powerful and widely utilized in habitat suitability modeling, their performance is strongly influenced by sampling bias, as pest occurrence data are often clustered, resulting in spatially biased predictions (Heikkinen et al. 2006; Phillips et al. 2009; Boria et al. 2014). Moreover, correlative ENM models fail to incorporate the underlying biological and physiological mechanisms that determine species distributions, thereby reducing their reliability when projecting responses under new climatic conditions.

On the other hand, mechanistic models such as DYMEX (Maywald et al. 2007) and ILCYM (Sporleder et al. 2017), among others, rely heavily on thermal tolerance and the life‐history traits of the species, which provide a process‐based understanding of species distribution. However, these models often require detailed biological data, such as life‐history traits obtained under controlled laboratory or field conditions, which are typically difficult and time‐consuming to generate (Maywald et al. 2007; Sporleder et al. 2017). In addition, mechanistic models mainly rely on temperature as the primary predictor variable, which simplifies the complex environmental interactions for the species (Maywald et al. 2007; Sporleder et al. 2017; Azrag et al. 2018). This narrow focus may overlook other critical abiotic factors, such as precipitation, which can strongly influence species establishment and survival. To overcome these limitations of either approach alone, a hybrid modeling framework that integrates both correlative and mechanistic methods was suggested to improve prediction accuracy (Agboka, Ndjomatchoua, Guimapi, et al. 2025). This approach leverages the predictive strength of correlative models and the mechanistic realism of process‐based models to produce more robust forecasts and potential distributions of species (Agboka, Ndjomatchoua, Guimapi, et al. 2025).

Despite the significant efforts in understanding the bioecology of RPW, there is a critical gap in developing predictive models under different climatic scenarios that could guide effective management and containment of this pest. Few studies have employed correlative ENM to predict the potential spread of RPW under current and future climate scenarios. For example, a recent study by Hayat et al. (2025) employed the Maxent algorithm to predict RPW in China, revealing a potential risk of expansion of the pest in the future. Additionally, the CLIMEX model predicted a significant increase in range expansion for RPW into northern China (Ge et al. 2015). However, these studies are localized and do not offer a comprehensive global overview of areas where RPW could establish, particularly in uninvaded regions where such information could guide targeted pest surveillance efforts. Fiaboe et al. (2012), in contrast, employed Maxent and GARP algorithms to predict RPW worldwide using bioclimatic variables. However, the future distribution of RPW under climate change remains unknown. Furthermore, depending on a single model for pest risk assessment introduces a degree of uncertainty, as each modeling approach has inherent limitations and assumptions that may not fully capture the complexity of pest distribution dynamics (Naimi et al. 2014; Shabani et al. 2016). Addressing these gaps through advanced modeling approaches for RPW would significantly enhance early detection, surveillance, and proactive management strategies for this important pest. Therefore, this study aimed to predict the potential distribution of RPW under current and future climatic conditions using a new modeling framework that integrates correlative and mechanistic approaches.

## 2 Methodology

**FIGURE 1:**
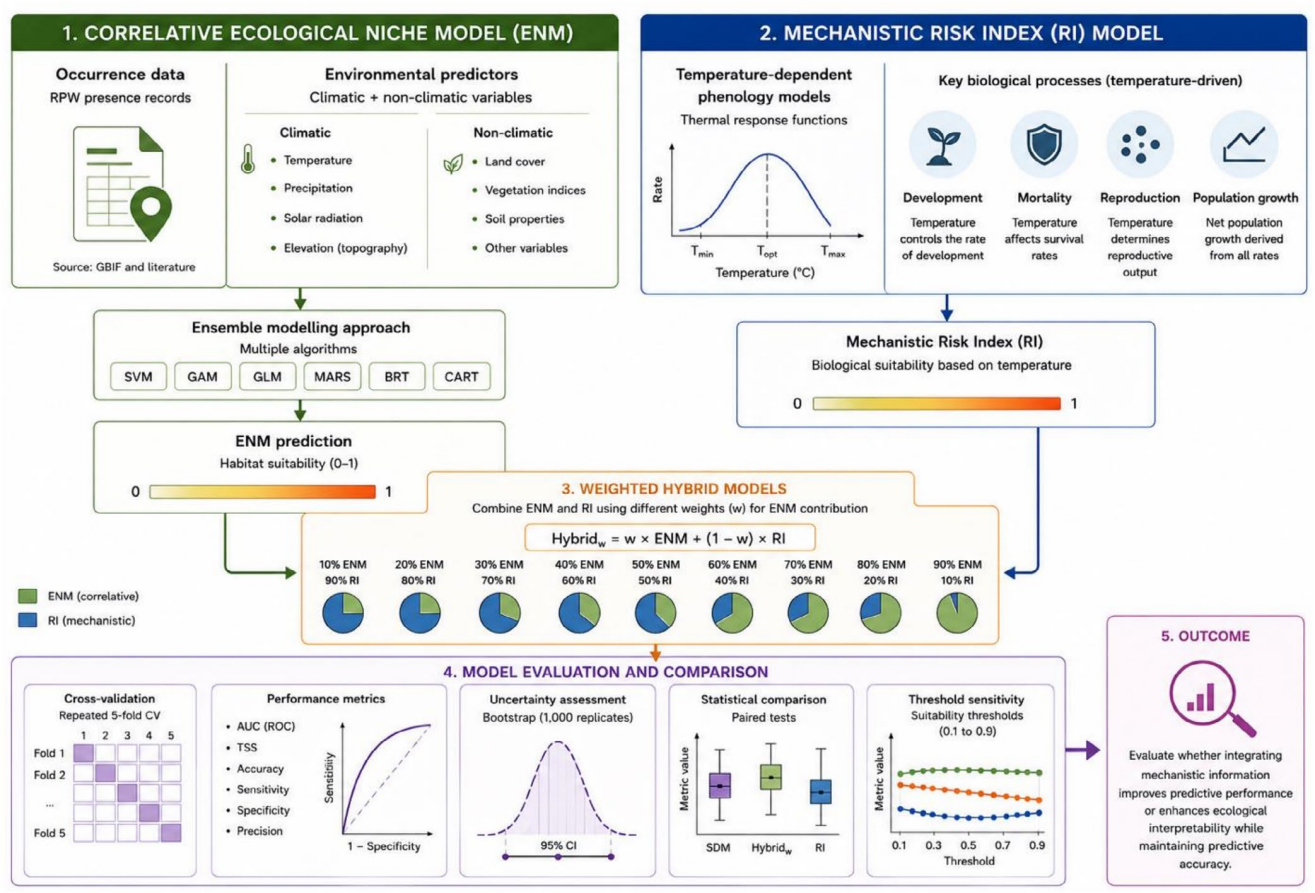
Schematic overview of the modeling framework used to evaluate the integration of correlative and mechanistic approaches for predicting the potential distribution of the red palm weevil (*Rhynchophorus ferrugineus*). The workflow comprises five stages: (1) development of a correlative ecological niche model (ENM) from occurrence records and environmental predictors; (2) construction of a temperature‐driven mechanistic Risk Index (RI) based on RPW phenology and life‐history traits; (3) generation of weighted hybrid models by combining the ENM and RI across different weighting schemes (10%–90% ENM contribution); (4) model evaluation using repeated five‐fold cross‐validation, bootstrap‐derived 95% confidence intervals, paired statistical comparisons, and threshold‐sensitivity analyses; and (5) assessment of whether mechanistic information improves predictive performance or enhances the ecological interpretability of species distribution models while maintaining predictive accuracy.

The methodology used in this study integrated a correlative ecological niche model (ENM) with a mechanistic Risk Index (RI) to evaluate alternative strategies for predicting the potential distribution of the red palm weevil (RPW) (Figure 1). The correlative ENM was developed using RPW occurrence records and environmental predictors, whereas the mechanistic RI was constructed from temperature‐dependent phenology models describing key biological processes, including development, mortality, reproduction, and population growth. The correlative model captured the empirical relationship between species occurrences and environmental conditions, representing the realized ecological niche shaped by climatic and non‐climatic factors. In contrast, the mechanistic RI quantified the biological suitability of the environment based on temperature, a major driver of insect development, survival, reproduction, and population dynamics (Sporleder et al. 2017; Azrag et al. 2018). To investigate how mechanistic information influences predictive performance, the ENM and RI were combined into a series of weighted hybrid models in which the contribution of the ENM ranged from 10% to 90% (Figure 1). Model performance was evaluated using repeated five‐fold cross‐validation and compared using the Area Under the Receiver Operating Characteristic Curve (AUC), True Skill Statistic (TSS), accuracy, sensitivity, specificity, and precision. Predictive uncertainty was quantified using bootstrap‐derived 95% confidence intervals, while paired statistical comparisons were used to evaluate differences among the SDM, RI, and hybrid models. The robustness of model performance was further assessed through threshold‐sensitivity analyses across a range of suitability thresholds. This framework enabled a rigorous evaluation of whether integrating mechanistic information improves predictive performance or instead enhances the ecological interpretability of species distribution models while maintaining predictive accuracy.

### 2.1 Correlative Ecological Niche Models (ENMs)

#### 2.1.1 RPW Occurrence Data

The worldwide distribution records of RPW, including 3355 observations from GBIF (GBIF 2024) and 1109 from iNaturalist https://www.inaturalist.org/ were obtained to model the potential distribution of RPW using correlative ENM. These georeferenced records represent locations where RPW has been observed. Before modeling, the occurrence records underwent a thorough cleansing process. This process involved the removal of duplicated records, erroneous coordinates, and spatial outliers. In addition, we used spThin package v0.2.028 (Aiello‐Lammens et al. 2015) in the R environment to spatially thin the occurrence records and retained only one record within 5 km^2^. This procedure ensures the highest data quality and minimizes potential biases and spatial autocorrelation. After cleaning and thinning, we retained 2742 occurrence records of RPW that were utilized to predict its potential distribution under current and future climatic conditions.

#### 2.1.2 Selection of Bioclimatic and Remotely Sensed Variables

**FIGURE 2:**
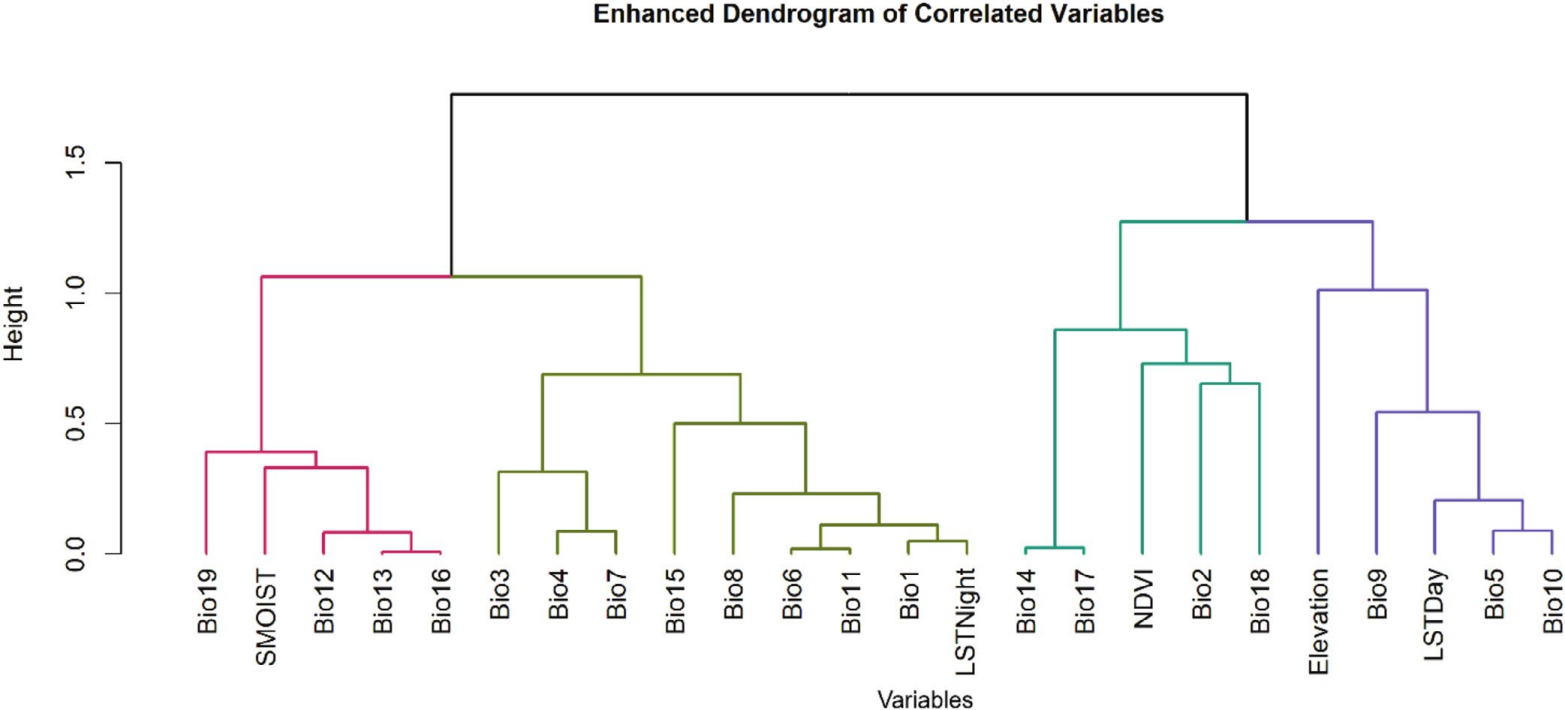
The enhanced correlation distance dendrogram among the predictor variables. The height of the branch point indicates the level of similarity between clusters, with shorter branch points indicating higher similarity between the variables (stronger correlation between the variables).

Bioclimatic predictors (19 variables) and one topographic variable (elevation) of WorldClim datasets were used as input layers to project the current distribution of RPW (Table 1) (Fick and Hijmans 2017). Although these layers produce sufficient output of the species habitat (Azrag et al. 2025), we also included remotely sensed variables, including normalized difference vegetation index (NDVI), land surface temperature during the day and night, and soil moisture, to increase the robustness of the correlative ENM model (Table 1). However, predictor variables often have a higher degree of collinearity with each other, which could affect model performance and produce misleading output if not properly handled (Heikkinen et al. 2006; Dormann et al. 2013; Van Tran et al. 2024). To address this, we used an enhanced correlation distance dendrogram to identify the correlated variables (Figure 2). This approach calculates the distance (height) as one minus the absolute value of the correlation coefficient (1 − |*r*|) and then clusters the variables in groups based on their correlation or similarity. We also used the Variance Inflation Factor (VIF) as a complementary metric to identify correlated variables, offering deeper insights beyond those provided by pairwise correlations (Naimi et al. 2014; Naimi and Araújo 2016). A variable with a VIF of 10 or higher was considered highly correlated and was therefore excluded from the modeling process. After this step, 14 uncorrelated variables remained, which were used to predict habitat suitability of RPW under current conditions (Table 1; Figure 2). To project the potential future distribution of RPW (2061–2080), we employed the same set of selected variables under two climate change scenarios, SSP2‐4.5 and SSP5‐8.5 (Fick and Hijmans 2017). SSP2‐4.5 represents an intermediate emissions scenario with moderate climate mitigation, whereas SSP5‐8.5 represents a high‐emissions scenario with limited mitigation and continued increases in greenhouse gas concentrations. These scenarios enabled assessment of the potential impacts of moderate and severe climate change on the future distribution and invasion risk of RPW.

**TABLE 1:** Bioclimatic and remotely sensed variables used to project the potential distribution of RPW.

Source	Variable category	Variables description	Unit	Abbreviation
WorldClim	Bioclimatic	Annual mean temperature	°C	Bio1
	
**Mean diurnal range (mean of monthly (max temp – min temp))**	**°C**	**Bio2**
	
**Isothermality (Bio2/Bio7) (× 100)**	**°C**	**Bio3**
	
Temperature seasonality (standard deviation ×100)	°C	Bio4
	
Max temperature of the warmest month	°C	Bio5
	
Min temperature of the coldest month	°C	Bio6
	
Temperature annual range (Bio 5 – Bio 6)	°C	Bio7
	
**The mean temperature of the wettest quarter**	**°C**	**Bio8**
	
**The mean temperature of the driest quarter**	**°C**	**Bio9**
	
The mean temperature of the warmest quarter	°C	Bio10
	
The mean temperature of the coldest quarter	°C	Bio11
	
Annual precipitation	mm	Bio12
	
**Precipitation of the wettest month**	**mm**	**Bio13**
	
**Precipitation of driest month**	**mm**	**Bio14**
	
**Precipitation seasonality (coefficient of variation)**	**mm**	**Bio15**
	
Precipitation of the wettest quarter	mm	Bio16
	
Precipitation of the driest quarter	mm	Bio17
	
**Precipitation of the warmest quarter**	**mm**	**Bio18**
	
**Precipitation of the coldest quarter**	**mm**	**Bio19**

Topographic	**Elevation**	**masl**	**Elevation**
MODIS	Remotely sensed	**Normalized difference vegetation index**	—	**NDVI**
USGS		**Land surface temperature during the day**	**°C**	**LSTDay**
		**Land surface temperature during the night**	**°C**	**LSTNight**
University of California Merced		**Soil moisture**	**mm**	**SMOIST**

*Note:* The uncorrelated variables in bold are the ones utilized in the final modeling process to predict the distribution of RPW.

#### 2.1.3 Ecological Niche Model Development

The occurrence records of RPW, along with the selected predictor variables, were integrated into the “*sdm*” package in the R software (Naimi and Araújo 2016) to predict the habitat suitability of RPW worldwide. Initially, we fitted 18 ecological niche models embedded in the “*sdm*” package to predict the potential distribution of RPW. Among these models, we selected the best‐fitted one based on the accuracy metrics, including the area under the curve of the receiver operating characteristic (AUC) and true skill statistics (TSS). We selected six best models, including support vector machines (SVM), generalized additive model (GAM), GLM, multivariate adaptive regression splines (MARS), boosted regression tree (BRT), and classification and regression tree (CART) to predict the potential distribution of RPW. Due to the potential variations in the outputs between different models using the same datasets (Naimi and Araújo 2016), we utilized the ensemble approach to maximize the prediction accuracy (Naimi and Araújo 2016; Guo et al. 2019). This approach combined the outputs of the six selected algorithms using the TSS weighted average. The ensemble approach provides superior predictive accuracy and greater overall reliability compared to individual model outputs (Naimi and Araújo 2016; Hao et al. 2020).

### 2.2 Mechanistic Species Distribution Models

To model the potential distribution of RPW using a mechanistic approach, we computed a Risk Index (RI) based on a set of ordinary differential equations (ODE) (Equation 1) for the population dynamics proposed by Rossini et al. (2021, 2022) and Ndjomatchoua et al. (2024). The RPW has four life stages: egg (e), larva (l), pupa (p) and adult (a), and their temperature‐dependent development, survival, and reproduction were described by Li et al. (2010). Based on this, we developed the corresponding population dynamics of RPW over time using the following system:

(1) $$\begin{array}{l} \frac{d}{\mathrm{dt}}e\left(t\right)=f\left(t\right)\times a\left(t\right)-\left[d_{1}\left(t\right)+m_{1}\left(t\right)\right]\times e\left(t\right)\\ \frac{d}{\mathrm{dt}}l\left(t\right)=d_{1}\left(t\right)\times e\left(t\right)-\left[d_{2}\left(t\right)+m_{2}\left(t\right)\right]\times l\left(t\right)\\ \begin{array}{c} \frac{d}{\mathrm{dt}}p\left(t\right)=d_{2}\left(t\right)\times l\left(t\right)-\left[d_{3}\left(t\right)+m_{3}\left(t\right)\right]\times p\left(t\right)\\ \frac{d}{\mathrm{dt}}a\left(t\right)=d_{3}\left(t\right)\times p\left(t\right)-m_{4}\left(t\right)\times a\left(t\right) \end{array} \end{array}$$

where $\;d_{1}\left(t\right)$, $d_{2}\left(t\right)$, and $d_{3}\left(t\right)$ are development rates from egg to larva, from larva to pupa and from pupa to adult, respectively, $m_{1}\left(t\right)$, $m_{2}\left(t\right)$,$m_{3}\left(t\right)$ and, $m_{4}\left(t\right)$ are temperature‐dependent mortality rates for egg, larva, pupa, and adult, respectively. The function $f\left(t\right),$ instead, denotes the temperature‐dependent fecundity rate of adult females, specifically female total oviposition, oviposition rate and sex ratio.

The transition rates into the model (1) are represented by specific functions listed in Table 2. The derived risk index (RI), the maximum eigenvalue of the Metzler matrix, indicates the temperature dependence of the transition rates within the model, where the numerator reflects growth potential via fecundity and development, while the denominator reflects mortality and developmental bottlenecks (Equation 2) (Agboka, Ndjomatchoua, Rossini, et al. 2025).

(2) $$\mathrm{RI}\left(t\right)=\frac{\left[f\left(t\right)\times d_{1}\left(t\right)\times d_{2}\left(t\right)\times d_{3}\left(t\right)\right]}{\left[d_{1}\left(t\right)+m_{1}\left(t\right)\right]\times \left[d_{2}\left(t\right)+m_{2}\left(t\right)\right]\times \left[d_{3}\left(t\right)+m_{3}\left(t\right)\right]\times m_{4}\left(t\right)}$$

and can be biologically interpreted as follows:

- $\mathrm{RI}\left(t\right)<1$: The insect population declines over time and is unlikely to establish.
- $\mathrm{RI}\left(t\right)=1$: The insect population size persists but not increase.
- $\mathrm{RI}\left(t\right)>1$: The insect population increases over time, and establishment is likely.

**TABLE 2:** Summary of temperature‐dependent trait functions used in computing the risk index (RI) of RPW.

Variables	Definition	Mathematical expression	Reference of data provenance
F	Fecundity rate function (eggs per female per sex ratio)	$1.75\exp\left(-0.5{\left(\frac{T-30.21}{3.98}\right)}^{2}\right)$	Li et al. (2010)
$d_{1}$	Development rate function for egg stage	$\left\{\begin{array}{c} 0.00226T\left(T-16.76\right)\sqrt{1-0.02T},\text{if}\;16.76<T<50\\ 0,\text{otherwise} \end{array}\right.$	Li et al. (2010)
$d_{2}$	Development rate function for larval stage	$\left\{\begin{array}{c} 4.65\times 10^{-5}\times T\left(T-16.56\right)\sqrt{1-0.02T},\text{if}\;16.56<T<50\\ 0,\text{otherwise} \end{array}\right.$	Li et al. (2010)
$\;d_{3}$	Development rate function for pupal stage	$\left\{\begin{array}{c} 8.73\times 10^{-5}\times T\left(T-14.77\right)\sqrt{1-0.02T},\text{if}\;14.77<T<50\\ 0,\text{otherwise} \end{array}\right.$	Li et al. (2010)
$m_{1}$	Mortality rate function for eggs	$\left(-0.00073\;T^{2}-0.0446T+0.72996\right)$	Li et al. (2010)
$\;m_{2}$	Mortality rate function for larvae	$\left(-0.00066\;T^{2}-0.0397T+0.65608\right)$	Li et al. (2010)
$\;m_{3}$	Mortality rate function for pupae	$\left(-0.00073\;T^{2}-0.0452T+0.71769\right)$	Li et al. (2010)
$m_{4}$	Mortality rate function for adults (inverse of the adult lifespan)	$\left(\;0.00006886401\;T^{2}-0.003194153T+0.04494223\right)$	Peng et al. (2016)

**FIGURE 3:**
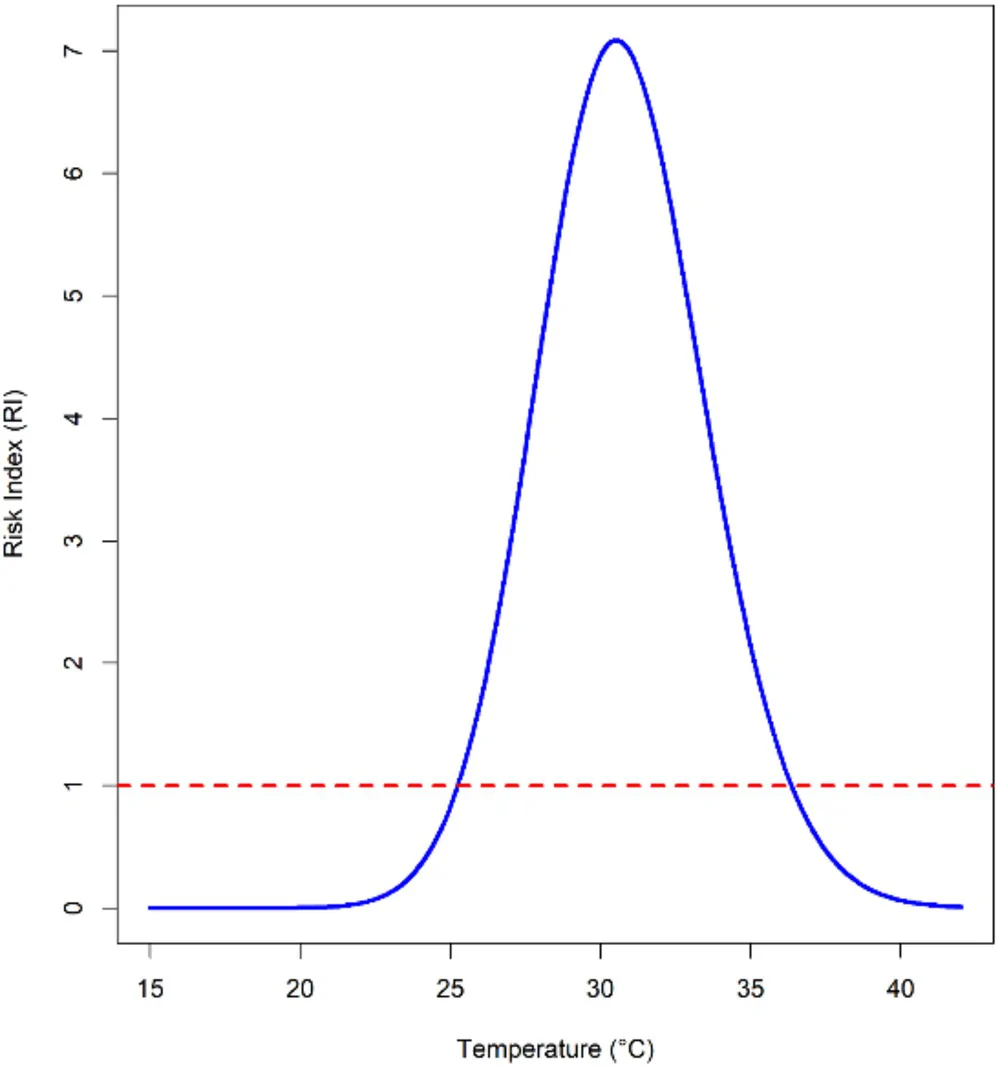
Computed risk index of RPW under temperature range using ordinary differential equations.

The risk index was computed in R software (Figure 3) and it assumed: (i) temperature is the only environmental parameter influencing RI of RPW, as the development, mortality, and reproduction are computed from average temperature values, with no intra‐seasonal variability, (ii) reflects long‐term stability, (iii) does not account for trophic relations between the host plant, the pest, and the natural enemies, (iv) assumes that the individuals of the population are exposed to the same temperature, neglecting microclimatic effects, (v) is parameterised via laboratory experiments, and (vi) the rate of variation is linearly dependent on the state variables, assuming the scalability of the information.

To map the risk index (RI) shown in Figure 3 (mechanistic distribution) for the red palm weevil (RPW), we incorporated temperature‐based bioclimatic variables known to influence the species' establishment and persistence using Equation 3. Specifically, we extracted the annual mean temperature (Bio1) and the mean temperature of the warmest quarter (Bio5) from global climate datasets, using the same present and future climate sources as those applied in the ecological niche modeling framework (Table 1). Bio1 represents long‐term average thermal conditions, whereas Bio5 captures seasonal thermal extremes that may impose physiological stress during critical life‐cycle stages. To approximate a more realistic thermal environment for establishment, we calculated the average of these two variables and used the resulting raster as the temperature input for RI mapping. This approach was preferred over a single mean temperature, as the RI is strongly influenced by temperature seasonality (Agboka, Ndjomatchoua, Guimapi, et al. 2025). The RI was computed across the spatial grid using this averaged temperature raster, applying temperature‐dependent functions that represent development rates, fecundity, and mortality. The spatial computation was implemented using the raster and terra packages in R.

To translate the continuous RI into an interpretable suitability probability, we applied the branching process–based transformation that estimates the probability that an introduced RPW would lead to a self‐sustaining outbreak (Equation 3) (Thompson et al. 2020; Agboka et al. 2026b):

(3) $$\mathrm{RI}=\left\{\begin{array}{cc} 0 & \mathrm{RI}\leq 1\\ 1-\frac{1}{\mathrm{RI}} & \mathrm{RI}>1 \end{array}\right.$$

To facilitate quantitative evaluation, the continuous RI was transformed into a probability of population establishment using the branching‐process formulation (Equation 3), yielding values bounded between 0 and 1. Under this formulation, RI values equal to or below one correspond to zero establishment probability, whereas values greater than one produce progressively increasing probabilities of persistence. This transformation converts the mechanistic estimate of population growth into an ecologically interpretable measure of establishment likelihood while preserving the biological persistence threshold (RI > 1). The transformed RI probabilities were extracted at the same presence and background locations used for the correlative models, and predictive performance was evaluated using the identical repeated cross‐validation and performance assessment framework described above.

### 2.3 Hybrid Suitability Model of RPW

To integrate correlative and mechanistic information, we constructed an additive hybrid suitability model by combining the species distribution model (SDM) with the independent phenology‐based Risk Index (RI), both normalized to the interval [0,1]. The RI raster was first resampled to match the spatial resolution, extent, and coordinate reference system of the SDM, allowing direct pixel‐wise integration. The hybrid suitability model was defined as follows:

(4) $$H=w\times \mathrm{SDM}+\left(1-w\right)\times \mathrm{RI}$$

where *w* represents the contribution of the correlative SDM (Equation 4).

The SDM weight (*w*) was systematically varied from 0.1 to 0.9 in increments of 0.1, generating nine hybrid models ranging from predominantly mechanistic (10% SDM, 90% RI) to predominantly correlative (90% SDM, 10% RI) (Figure 1). This incremental weighting strategy follows the hybrid integration framework proposed by Agboka et al. (2026a), providing a standardized approach for quantifying how increasing contributions of correlative and mechanistic information influence predictive performance.

### 2.4 Model Performance

Model performance was evaluated using the same presence and randomly generated background datasets for all models. Suitability values were extracted from the SDM, RI, and each hybrid raster at all evaluation points, and repeated five‐fold cross‐validation (20 repetitions) was performed to obtain robust estimates of predictive performance. For each training dataset, the optimal classification threshold was determined using the Youden index derived from receiver operating characteristic (ROC) analysis, and this threshold was subsequently applied to the corresponding testing dataset. Model performance was quantified using the Area Under the Receiver Operating Characteristic Curve (AUC), True Skill Statistic (TSS), overall accuracy, sensitivity, specificity, and precision.

To quantify predictive uncertainty, 1000 bootstrap resamples of the evaluation dataset were generated for each model. Performance metrics were recalculated for every bootstrap replicate, and percentile‐based 95% confidence intervals were derived for all evaluation metrics. Statistical differences between the SDM, RI, and the best‐performing hybrid model were assessed using paired Wilcoxon signed‐rank tests across repeated cross‐validation folds. Finally, the robustness of model performance was evaluated through threshold‐sensitivity analyses by recalculating classification metrics across suitability thresholds ranging from 0.1 to 0.9. The hybrid model exhibiting the highest mean cross‐validated TSS was retained as the optimal integration strategy for subsequent spatial prediction and comparison.

## 3 Results

### 3.1 The Ensemble Ecological Niche Model for RPW

**FIGURE 4:**
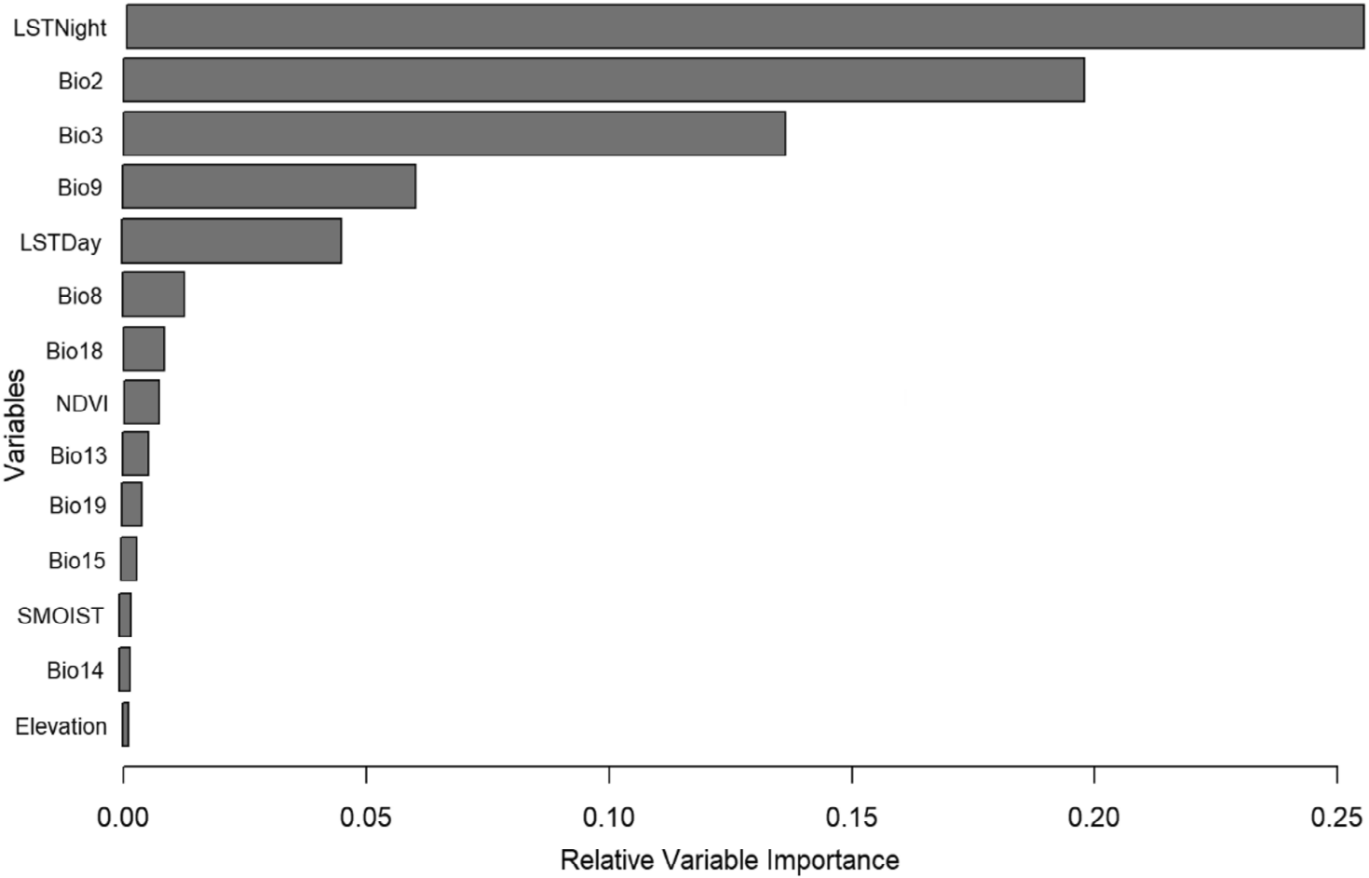
Relative importance of the predictor variables in predicting the potential distribution of RPW using an ensemble modeling approach, based on the weighted average performance of six machine‐learning algorithms: Support vector machines (SVM), generalized additive model (GAM), generalized linear model (GLM), multivariate adaptive regression splines (MARS), boosted regression tree (BRT), and classification and regression tree (CART).

The ensemble ecological niche model (ENM), integrating six correlative algorithms (SVM, GAM, GLM, MARS, BRT, and CART), demonstrated excellent predictive performance (Table 3). Repeated five‐fold cross‐validation yielded a mean AUC of 0.984 ± 0.002 and a TSS of 0.891 ± 0.009, together with an overall accuracy of 0.946 ± 0.004. The ensemble model also achieved high sensitivity (0.955 ± 0.007), specificity (0.936 ± 0.009), and precision (0.715 ± 0.007), indicating a robust ability to discriminate suitable from unsuitable environments for RPW establishment. Among the variables that were selected for the ENM, land surface temperature during night (LSTNight), mean diurnal range (Bio2), isothermality (Bio3), and the mean temperature of the driest quarter (Bio9) were the most important variables in predicting the habitat suitability of RPW, with contributions of 25.5%, 19.8%, 13.6%, and 6%, respectively (Figure 4).

**TABLE 3:** Cross‐validated performance of the mechanistic Risk Index (RI), optimal hybrid model (Hybrid_90), and correlative SDM.

Metric	SDM	RI	Hybrid_90
AUC	0.984 ± 0.002	0.755 ± 0.009	0.983 ± 0.002
TSS	0.891 ± 0.009	0.457 ± 0.017	0.893 ± 0.009
Accuracy	0.946 ± 0.004	0.649 ± 0.022	0.897 ± 0.007
Sensitivity	0.955 ± 0.007	0.824 ± 0.029	0.954 ± 0.007
Specificity	0.936 ± 0.009	0.633 ± 0.026	0.939 ± 0.004
Precision	0.715 ± 0.007	0.174 ± 0.008	0.589 ± 0.007

**FIGURE 5:**
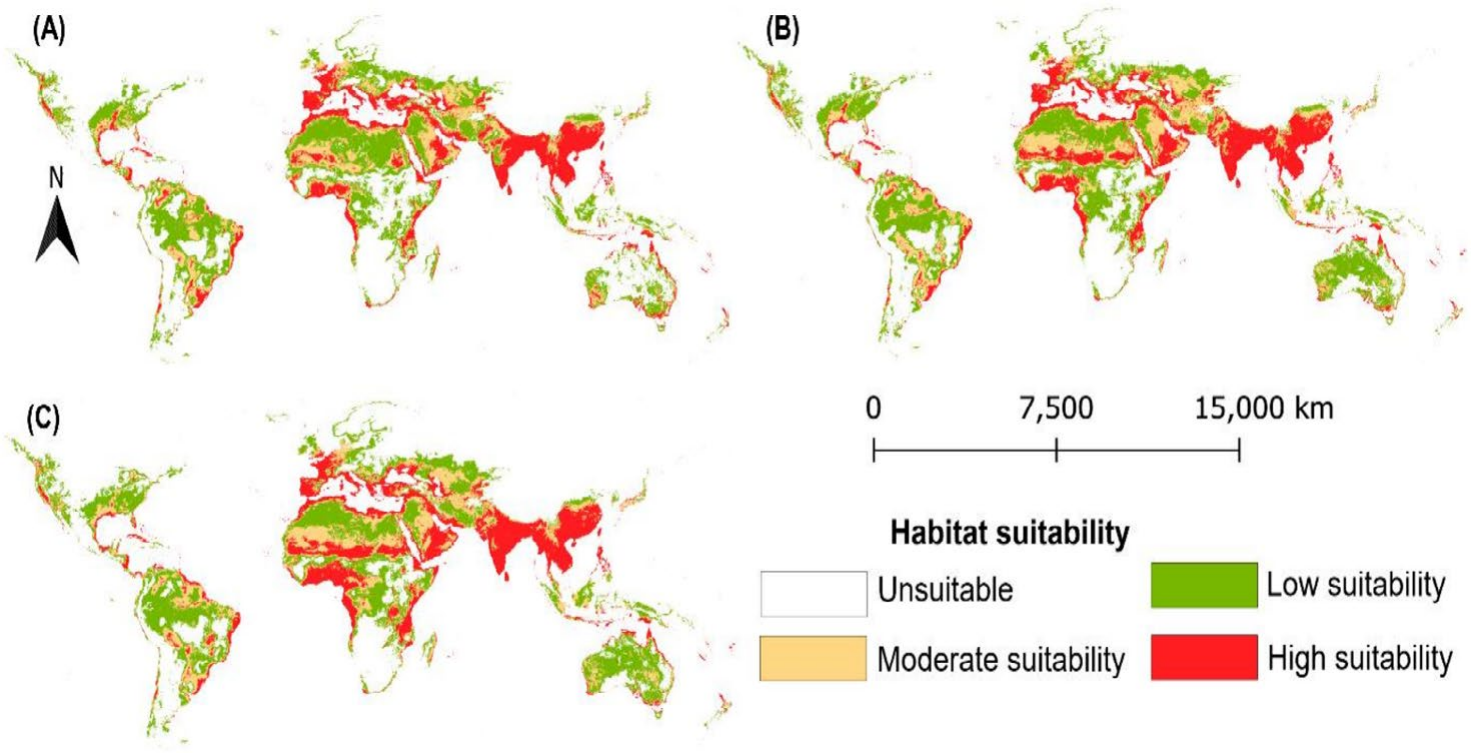
Potential distribution of the red palm weevil (RPW) under (A) current climatic conditions, (B) future climatic conditions projected under the SSP2–4.5 scenario, and (C) future climatic conditions projected under the SSP5–8.5 scenario. Predictions were generated using an ensemble model integrating six machine‐learning algorithms: Support vector machines (SVM), generalized additive model (GAM), generalized linear model (GLM), multivariate adaptive regression splines (MARS), boosted regression tree (BRT) and classification and regression tree (CART).

Under current climatic conditions, the ENM model identified areas of high suitability in Southeast Asia, the pest's region of origin (Figure 5A). Additionally, extensive suitable habitats were predicted across North Africa, the Middle East, and the Mediterranean Basin, consistent with the pest's known distribution. Coastal regions along the Indian Ocean, including Djibouti, Somalia, Kenya, Tanzania, Mozambique, and South Africa, were also predicted to provide highly suitable environments for RPW establishment. Similarly, coastal regions along the Atlantic Ocean in Africa, Europe, South America, and North America were identified as highly suitable under current climatic conditions (Figure 5A).

Future projections revealed an increase in suitable habitat under different climate change scenarios. Under the SSP2‐4.5 scenario, all currently suitable regions are expected to remain favorable for the pest to thrive, with increased suitability predicted particularly in West and Central Africa (Figure 5B). A similar trend was predicted under the high‐emission SSP5‐8.5 scenario, where the Middle East, Central, West, and East Africa regions are predicted to persist as hotspots for RPW to thrive (Figure 5C).

### 3.2 Risk Index for RPW

The mechanistic Risk Index (RI), derived exclusively from temperature‐dependent life‐history processes, demonstrated a moderate ability to discriminate suitable from unsuitable environments for RPW establishment (Table 3). Repeated five‐fold cross‐validation yielded a mean AUC of 0.755 ± 0.009 and a TSS of 0.457 ± 0.017, indicating that the RI successfully captured the fundamental thermal constraints governing RPW persistence but exhibited lower discriminatory power than the correlative SDM. The RI achieved an overall accuracy of 0.649 ± 0.022, with relatively high sensitivity (0.824 ± 0.029) but lower specificity (0.633 ± 0.026) and precision (0.174 ± 0.008), suggesting that the mechanistic model effectively identified climatically suitable environments while tending to overpredict areas where additional ecological or anthropogenic factors may limit establishment.

**FIGURE 6:**
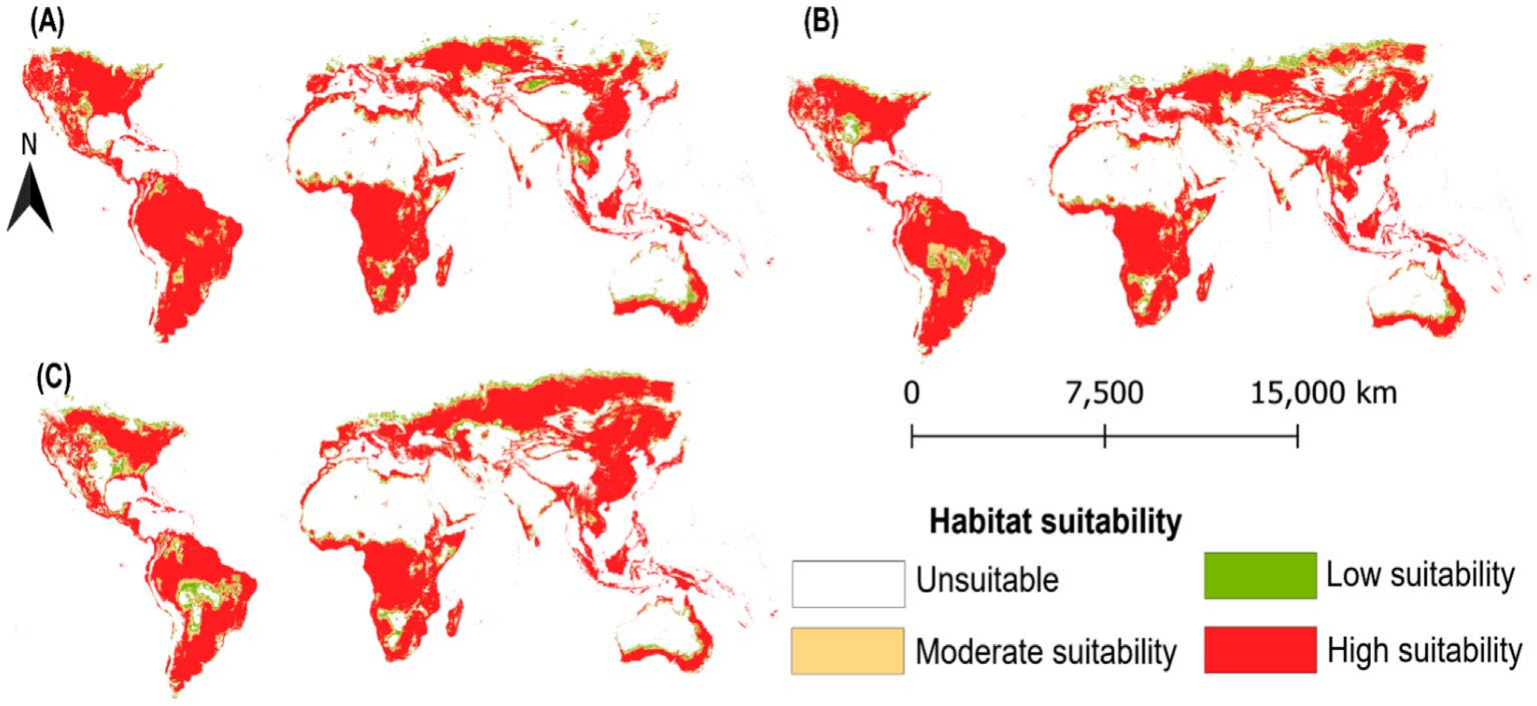
Potential distribution of RPW under (A) current climatic conditions, (B) future climatic conditions projected under the SSP2–4.5 scenario, and (C) future climatic conditions projected under the SSP5–8.5 scenario. Predictions were generated using a risk index calculated from temperature‐dependent development models for RPW.

Under current climatic conditions, the models indicated high risk areas for establishment in North Africa and sub‐Saharan Africa (Figure 6A). Similarly, a high potential for RPW establishment was predicted in the Mediterranean regions and some parts of the Middle East, where date palms are intensively cultivated (Figure 6A). The models also identified the USA, as well as Central and South America, as highly suitable regions for RPW establishment under the current climate scenario. However, despite predicting high suitability in parts of the Mediterranean region, the model underestimated RPW presence in Saudi Arabia, Yemen, the United Arab Emirates, and India compared to the predictions of the ensemble ecological niche model (Figure 6A).

Under future climatic conditions, the risk index model projects that large portions of Africa, South and Southeast Asia, Australia, and South America will remain highly suitable for RPW establishment and persistence, regardless of the climatic scenario (Figure 6B,C). However, the model underestimated habitat suitability in parts of the Middle East, where the pest is already known to occur.

### 3.3 Hybrid Prediction

**FIGURE 7:**
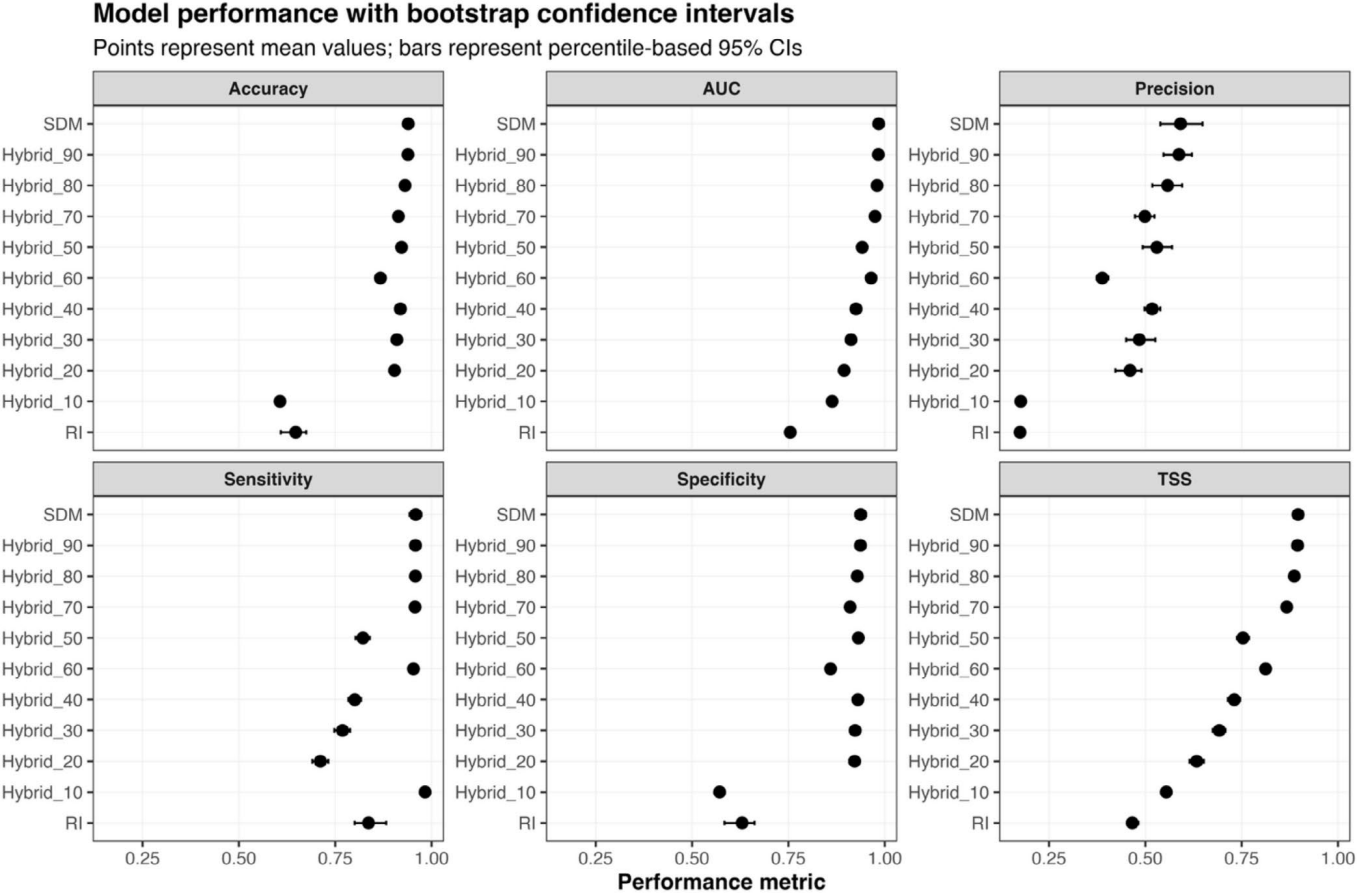
Best‐performing additive hybrid model identified through iterative weighting of mechanistic (phenology‐based RI) and correlative (SDM) components.

The optimal hybrid model (90% SDM and 10% RI; Figure 7), which integrated the ensemble ecological niche model with the temperature‐dependent RI through a weighted additive framework, achieved predictive performance comparable to that of the correlative SDM (AUC = 0.983 ± 0.002, TSS = 0.893 ± 0.009, sensitivity = 0.954 ± 0.007) (Table 3). Although it did not improve discrimination accuracy over the SDM alone, it substantially outperformed the mechanistic RI and retained high predictive performance while incorporating biologically meaningful information on species persistence. Paired Wilcoxon signed‐rank tests showed significant differences between Hybrid90 and the SDM for AUC, accuracy, precision, sensitivity, and specificity (all *p* < 0.0001), as well as TSS (*p* = 0.0383). At the same time, comparisons between Hybrid90 and the RI were significant across all evaluation metrics (all *p* < 0.0001).

**FIGURE 8:**
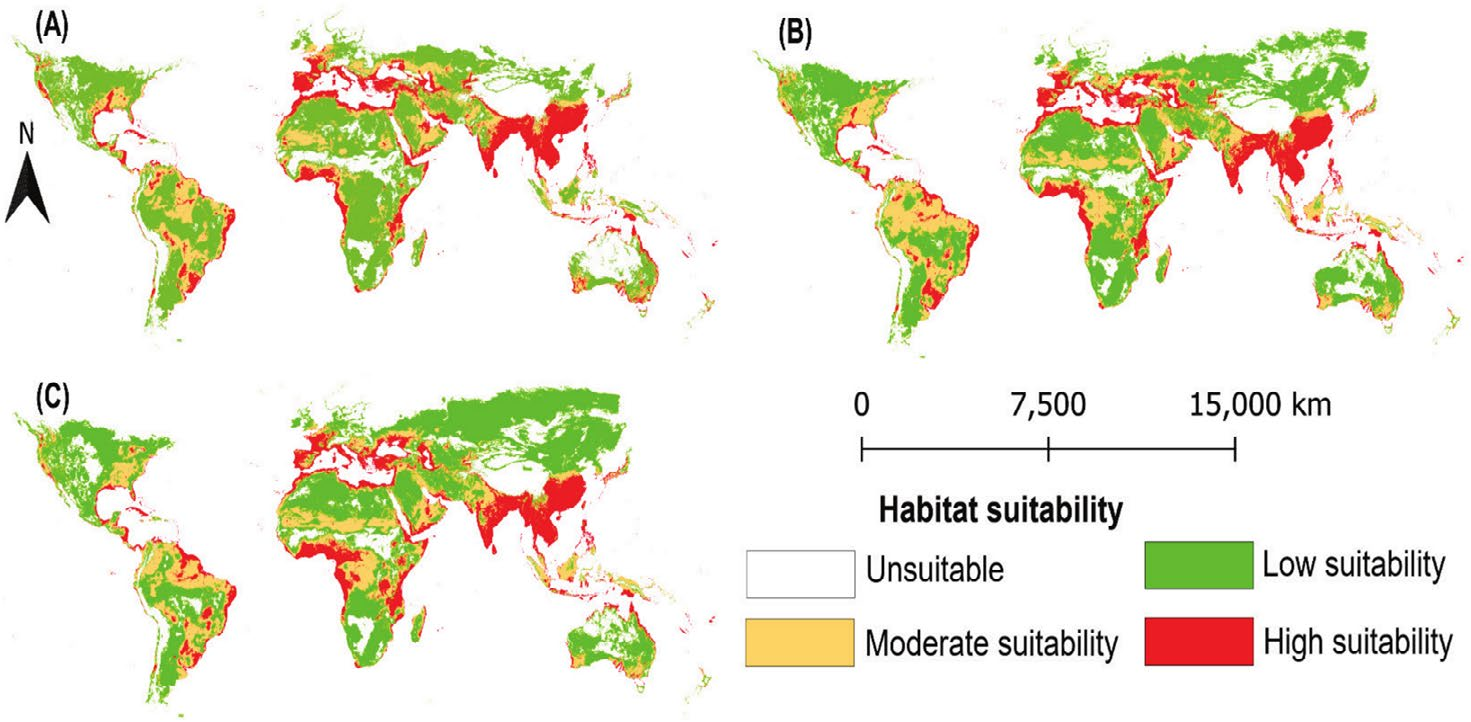
Potential distribution of RPW under (A) current climatic conditions, (B) future climatic conditions projected under the SSP2–4.5 scenario, and (C) future climatic conditions projected under the SSP5–8.5 scenario. Predictions were generated using a combination of the ensemble ecological niche model and the temperature‐dependent risk index.

The model accurately captured the current known distribution of RPW under present climatic conditions, particularly across South and Southeast Asia, the Middle East, and the Mediterranean region (Figure 8A). Additionally, West African countries along the Atlantic coast exhibited high habitat suitability for RPW establishment under current conditions. In Eastern Africa, countries such as Eritrea, Djibouti, Somalia, Kenya, Tanzania, and Uganda were also identified as high‐risk areas for invasion (Figure 8A). Beyond Africa, the southeastern and western parts of the United States, as well as regions in Central and South America, were projected to be suitable for RPW establishment.

Under future climatic conditions represented by the SSP2–4.5 scenario, the invasion risk of RPW is projected to expand across Africa, particularly in the northern, western, central, and eastern regions (Figure 8). Similarly, the risk is expected to intensify in countries such as Saudi Arabia, the United Arab Emirates, Kuwait, Iran, and Iraq. In the Americas, the suitable habitats for RPW are projected to extend further into the southern parts of the United States and across Central and South America. Under the same scenario, several Mediterranean countries in Europe, where RPW is currently established, are predicted to remain suitable for its survival (Figure 8B).

Under the high‐emission SSP5–8.5 scenario, countries in North Africa, the Mediterranean region, and parts of the Middle East, West, and Eastern Africa will likely remain highly suitable for RPW establishment and persistence (Figure 8C). However, the risk is projected to be higher in East, West, and Central Africa.

## 4 Discussion

In this study, we used a new modeling framework that combined an ensemble ecological niche model (ENM) generated from the pest occurrence data with a temperature‐dependent risk index (RI) to predict the potential distribution of RPW. While the ecological niche model predicted habitat suitability by correlating known occurrence records of RPW with environmental variables, the prediction of the risk index considered the physiological responses of the pest to temperature, reflecting its survival, development, reproduction, and population growth rate. The hybrid modeling framework therefore enhanced the ecological interpretability of species distribution predictions without compromising predictive performance. Although the correlative SDM remained the most accurate predictor, integrating a modest contribution of mechanistic information (10%) produced statistically comparable predictive performance while explicitly incorporating the thermal constraints governing RPW establishment and population persistence. Consequently, the hybrid model not only identified areas that are environmentally suitable for RPW occurrence but also incorporated biologically meaningful information on climatic conditions that support long‐term population establishment. Rather than improving discrimination accuracy, the integration of mechanistic and correlative information strengthened the ecological realism of the predictions, providing a more informative framework for invasion‐risk assessment and decision‐making for invasive insect pests whose development, survival, reproduction, and population dynamics are strongly regulated by temperature.

The ENM demonstrated excellent predictive performance, confirming its robustness for predicting the potential distribution of RPW and supporting previous studies demonstrating the strong performance of ensemble species distribution models for habitat suitability assessment (Shabani et al. 2016; Naimi and Araújo 2016; Becker et al. 2020). This indicates that the model effectively discriminated between suitable and unsuitable habitats, providing a good estimation of the RPW ecological niche. The land surface temperatures, mean diurnal temperature range (Bio2), and isothermality (Bio3) were the most important variables contributing to predicting the suitability of RPW using ensemble ENM. Bio2 and Bio3 highlight the importance of temperature variability in shaping RPW distribution. Bio2 reflects daily temperature fluctuations, which can influence development, survival, reproduction, and adult activity. Bio3 represents diurnal temperature variation relative to annual temperature variation and therefore reflects thermal stability and seasonality. Together, these variables suggest that both daily temperature fluctuations and longer‐term thermal stability influence the climatic suitability and potential distribution of RPW. On the other hand, night‐time land surface temperature (LSTNight) likely plays an important role in shaping the distribution of RPW because it reflects the minimum thermal conditions experienced during the weevil's primary period of activity. Adult RPW is predominantly crepuscular and nocturnal, with flight, mating, and host‐seeking occurring mainly during the evening and night (Faleiro 2006). Warmer night‐time temperatures can therefore enhance adult activity, dispersal, and oviposition, while also promoting faster larval development. Consequently, LSTNight provides ecologically meaningful information beyond conventional air temperature variables. Our findings corroborate earlier studies that emphasized temperature being the primary factor influencing RPW distribution and population dynamics (Hoddle et al. 2015; de Souza et al. 2025). Being a thermophilic insect, RPW thrives in regions with high temperatures, with lower and upper thermal thresholds reported at 15°C and 40°C, respectively (Li et al. 2010; Dembilio and Jacas 2011). This indicates that areas with high or moderate land surface temperature, such as coastal zones and palm plantations, provide suitable microclimates for RPW populations to thrive.

The ENM model prediction under current climatic conditions is well aligned with the currently known distribution range of RPW, especially in Southeast Asia, North Africa, the Middle East, and the Mediterranean Basin. Our results are consistent with the predictions of Fiaboe et al. (2012), who identified these areas as highly suitable for the establishment and persistence of RPW using correlative maxent and GARP algorithms. In addition, our model prediction in South America is in line with the findings of de Souza et al. (2025), who predicted the coastal regions of the Northeast, Southeast, and South of Brazil to be highly environmentally suitable for RPW to occur. Indeed, RPW establishment in coastal regions of Brazil has been confirmed (de Souza et al. 2025), lending strong support to the accuracy of our predictions. Beyond the currently known distribution of RPW, the ENM predicted suitable areas in North, West, and East Africa, the Southern and Western parts of the United States, coastal regions of South America, and Australia. Notably, RPW was recently detected in the Canary Islands in Spain and Djibouti in East Africa (FAO 2020), where our model also predicted high suitability for pest establishment, further confirming the accuracy of our model. Correlative ENMs are data‐driven and often provide better predictions in areas where occurrence records are available (Phillips et al. 2009). However, these models often underperform in unsampled regions where there are no occurrence records, as they capture only the environmental conditions presented within the calibration data, resulting in unreliable extrapolations (Elith and Leathwick 2009; Elith et al. 2010). This is because correlative models do not incorporate physiological constraints of the species like development, survival, and reproduction.

On the other hand, the mechanistic Risk Index (RI) successfully identified many of the major regions already invaded by RPW but exhibited substantially lower predictive performance than the correlative ENM. This suggests that the RI effectively captured the fundamental thermal requirements for RPW establishment but tended to overpredict climatically suitable areas because it did not account for other environmental and anthropogenic factors that shape the species' realized distribution (Ndjomatchoua et al. 2024; Agboka, Ndjomatchoua, Rossini, et al. 2025). These limitations likely contributed to the discrepancies observed between the RI and ENM predictions. Moreover, the RI does not account for phenotypic plasticity or local adaptation, which may have contributed to its underestimation of RPW suitability in parts of Southeast Asia and the Middle East, where populations may exhibit divergent thermal responses following adaptation to local environmental conditions. Hybrid modeling approaches that integrate correlative species distribution models with mechanistic models are increasingly recognized as providing more ecologically realistic predictions by combining statistical relationships with species' physiological constraints (Kearney and Porter 2009; Buckley et al. 2010).

In this study, the optimal hybrid model (90% SDM and 10% RI) achieved predictive performance comparable to that of the correlative SDM while substantially outperforming the mechanistic RI alone. These findings indicate that incorporating a modest contribution of mechanistic information preserves the excellent discrimination ability of the correlative SDM while explicitly accounting for the thermal constraints governing RPW establishment and persistence. Our results are consistent with those of Agboka et al. (2026a), who similarly demonstrated that integrating mechanistic and correlative information can enhance the ecological interpretability of species distribution models while maintaining high predictive performance. Comparable findings have also been reported for other economically important insects. For example, mechanistic–correlative integration has improved predictions of the potential distributions of the fall armyworm, *Spodoptera frugiperda* , by accounting for temperature‐dependent development and survival while maintaining the high predictive power of correlative models (Early et al. 2018). Similarly, Pertierra et al. (2020) demonstrated that combining physiological thresholds with climatic suitability substantially enhanced global invasion risk assessments for invasive species. These studies collectively suggest that hybrid approaches provide more realistic estimates of invasion potential than either modeling framework alone, particularly for species whose establishment is strongly constrained by temperature.

In our study, the integrated model effectively delineated the current global distribution of RPW, accurately reflecting its known occurrence in South and Southeast Asia, the Middle East, and the Mediterranean Basin (Azmi et al. 2017; Al‐Dosary et al. 2016; Nurashikin‐Khairuddin et al. 2022). These regions represent the pest's native and long‐established invasive ranges, confirming the model's reliability in replicating observed patterns. However, the hybrid model underestimated pest occurrence in some regions such as Saudi Arabia, where the RI projected relatively low suitability. This highlights the importance of using high‐quality, geographically representative life‐history trait data when computing the RI, as regional variation in pest responses may influence model predictions. Under future climatic conditions, our predictions showed substantial expansion of invasion risk in Africa, especially in the Western, Central, and Eastern parts of the continent. However, this invasion risk will be high under the high emission scenario (SSP5–8.5), compared to the moderate scenario SSP2–4.5. This expansion might be attributed to an increase in temperature regimes that may accelerate the development and fecundity of RPW, leading to increased capacity of establishment in new environments. Similar climate‐driven expansions have been predicted for several other invasive pests, highlighting the broader ecological implications of warming climate trends in global invasion (Zhu et al. 2017; Zingore et al. 2020; Azrag et al. 2022). An increase in RPW risk was also predicted in the Arabian Peninsula, where date palm is extensively cultivated, reflecting the favorable agroclimatic conditions for the pest to thrive. In contrast, several Mediterranean countries in Europe, where RPW is currently established, are projected to become less suitable under this scenario, likely due to rising temperatures exceeding the pest's upper thermal tolerance limits.

Our prediction outcomes have significant implications for the prevention and spread of RPW in new areas under changing climatic conditions. Our model showed the invasive potential of RPW beyond its current range, especially in tropical and subtropical regions where extensive coconut, date, and oil palms are cultivated. These findings are critical for designing targeted monitoring, quarantine, and management strategies, especially in areas that are currently free of RPW but predicted to be environmentally suitable. For example, the coastal region of the Indian Ocean, where coconut is extensively grown, is predicted to be highly suitable for RPW establishment. Therefore, the presence of RPW in Djibouti poses a significant threat to Somalia, Kenya, and the entire East African region unless strong phytosanitary measures and continuous surveillance are undertaken to prevent the introduction and establishment of the pest in uninvaded areas. Similarly, the confirmed establishment of RPW in Brazil represents a significant biosecurity threat to neighboring countries, given the species' high dispersal capacity and the intensity of regional trade and movement of infested plant material. Strengthening phytosanitary regulations, quarantine inspections, and public awareness campaigns will be crucial to mitigating the spread. In the invaded regions, such as the Mediterranean Basin and the Middle East, our prediction showed high climate suitability for RPW persistence. Therefore, there is a need for adaptive management strategies that integrate biological control using parasitoids, biopesticides, pheromone‐based trapping, monitoring, and good agricultural practices to suppress RPW populations under varying climatic conditions.

## 5 Conclusion

Integrating the ecological niche model (ENM) with the temperature‐based Risk Index (RI) demonstrated that mechanistic information can enhance the ecological interpretability of species distribution models without compromising predictive performance. Although the correlative ENM remained the most accurate predictor, it achieved statistically comparable performance while explicitly incorporating the thermal constraints governing RPW establishment and persistence. The hybrid framework successfully reproduced the known global distribution of RPW and identified additional regions environmentally and biologically suitable for future invasion across Africa, the Middle East, and the Americas. Future climate projections, particularly under SSP5‐8.5, indicate substantial expansion of suitable areas in West, Central, and East Africa and the Arabian Peninsula, whereas parts of the Mediterranean Basin may become less suitable as temperatures exceed the upper thermal limits of the species. These findings highlight the importance of integrating mechanistic knowledge with correlative modeling to support biologically informed invasion‐risk assessment, strengthen surveillance and phytosanitary measures in regions projected to become suitable, and improve proactive management of invasive pests under climate change.

## Author Contributions

**Abdelmutalab G. A. Azrag:** conceptualization (equal), data curation (equal), formal analysis (equal), investigation (equal), methodology (equal), software (equal), validation (equal), visualization (equal), writing – original draft (equal), writing – review and editing (equal). **Komi Mensah Agboka:** conceptualization (equal), data curation (equal), formal analysis (equal), investigation (equal), methodology (equal), software (equal), validation (equal), visualization (equal), writing – review and editing (equal). **Elfatih M. Abdel‐Rahman:** conceptualization (equal), investigation (equal), methodology (equal), supervision (equal), validation (equal), visualization (equal), writing – review and editing (equal). **Rehemah Gwokyalya:** conceptualization (equal), investigation (equal), methodology (equal), validation (equal), visualization (equal), writing – review and editing (equal). **Seid A. Kemal:** conceptualization (equal), methodology (equal), supervision (equal), validation (equal), visualization (equal), writing – review and editing (equal). **Hamadttu A. F. El‐Shafie:** conceptualization (equal), investigation (equal), supervision (equal), validation (equal), writing – review and editing (equal). **Mohamed A. Bob:** conceptualization (equal), investigation (equal), methodology (equal), validation (equal), writing – review and editing (equal). **Samira A. Mohamed:** conceptualization (equal), funding acquisition (equal), investigation (equal), methodology (equal), project administration (equal), supervision (equal), validation (equal), writing – review and editing (equal). **Sunday Ekesi:** conceptualization (equal), funding acquisition (equal), investigation (equal), methodology (equal), supervision (equal), validation (equal), writing – review and editing (equal). **Abdou Tenkouano:** conceptualization (equal), funding acquisition (equal), investigation (equal), project administration (equal), resources (equal), supervision (equal), validation (equal), writing – review and editing (equal).

## Funding

This study was supported by the Gates Foundation and the United Arab Emirates government through the International Consortium for the Control of the Red Palm Weevil (C4RPWC), under project number C4RPWC‐INV‐080101.

## Conflicts of Interest

The authors declare no conflicts of interest.

## Data Availability

The data and code that support the findings of this study are publicly available in the repository of the International Centre of Insect Physiology and Ecology (*icipe*) at the following link: https://dmmg.icipe.org/dataportal/dataset/from‐suitability‐to‐invasion‐risk.
